# Ruptured Diaphragmatic Eventration: Lessons Learned

**DOI:** 10.7759/cureus.71929

**Published:** 2024-10-20

**Authors:** Nikhil Krishnani, Priya Gupta, Roysuneel Patankar

**Affiliations:** 1 Department of Surgery, Zen Hospital, Mumbai, IND; 2 Department of Gastrosurgery, Zen Hospital, Mumbai, IND

**Keywords:** diaphragm eventration, diaphragm plication, laparoscopic approach, ruptured diaphragmatic eventration, thoracoscopic approach

## Abstract

Diaphragmatic eventration (DE) is characterized by abnormal diaphragm elevation resulting from muscle weakness or thinning. It may be congenital or acquired, affecting both pediatric and adult populations, and is associated with symptoms such as dyspnea and chest pain. Although DE is infrequent, with a higher incidence on the left side, it has traditionally been managed through invasive surgical techniques. Advances in endoscopic methods, including thoracoscopic and laparoscopic plication, have revolutionized treatment by offering reduced postoperative pain, faster recovery, and improved precision. Despite these benefits, potential complications such as pneumothorax and diaphragm rupture remain concerns. We report two cases of ruptured DE and their management. Effective management requires meticulous surgical technique and consideration of individual patient factors to optimize outcomes and minimize complications.

## Introduction

The diaphragm, a dome-shaped muscle that separates the thoracic and abdominal cavities, is essential for inspiratory phase of breathing. The phrenic nerve innervates it. When the diaphragm fails to contract effectively while maintaining its anatomical attachments, it can elevate abnormally, partially or fully. This condition, known as diaphragmatic eventration (DE), results from a compromised diaphragm due to weakness and thinning. DE can be either congenital or acquired, affecting both adults and children. Patients with DE may present with symptoms such as dyspnea, upper abdominal pain, and chest pain, which vary depending on the severity. The incidence of DE is very low, less than 0.05% [1], and it is notably more common in the left hemidiaphragm, up to eight times more frequent on the left side in males [2]. Historically, DE was managed through thoracotomy or laparotomy for diaphragm plication. However, advancements in endoscopic surgery have provided a quicker and safer treatment option. Endoscopic techniques offer several advantages, including reduced pain, faster recovery, smaller incisions, and more precise treatment. Despite these advancements, the literature on ruptured DE remains limited.

This report presents two cases of ruptured DE managed by minimally invasive surgery.

## Case presentation

Case 1

**Figure 1:**
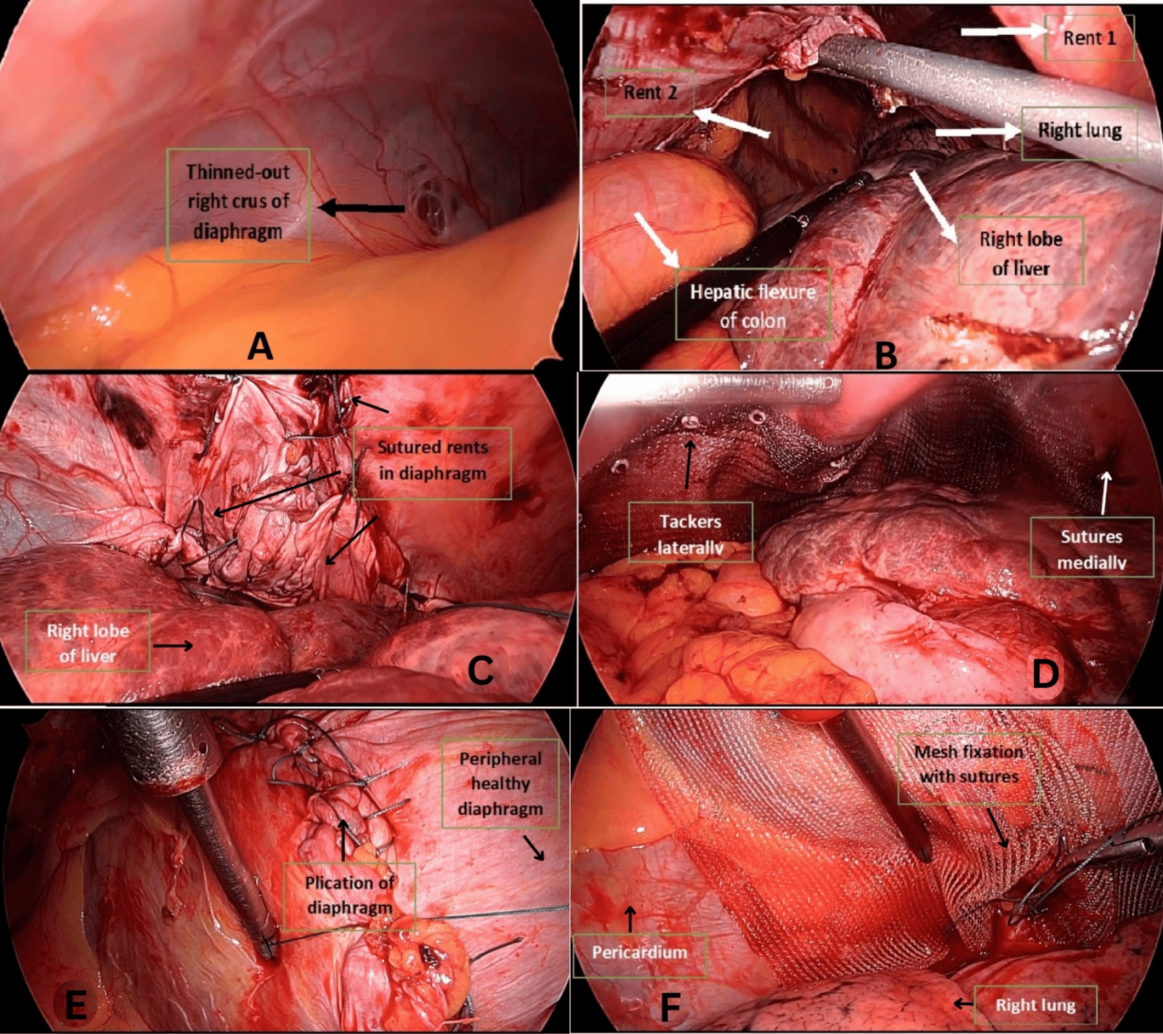
A) Right dome showing a thinned-out diaphragm. B) Two defects with herniation of abdominal contents into the right hemithorax. C) Laparoscopic closure of defects. D) Placement of the mesh and fixation with tackers and sutures on the abdominal side of the diaphragm. E) Thoracoscopic plication of the right dome of the diaphragm. F) Placement of the mesh and fixation with sutures on the thoracic side of the diaphragm.

**Figure 2:**
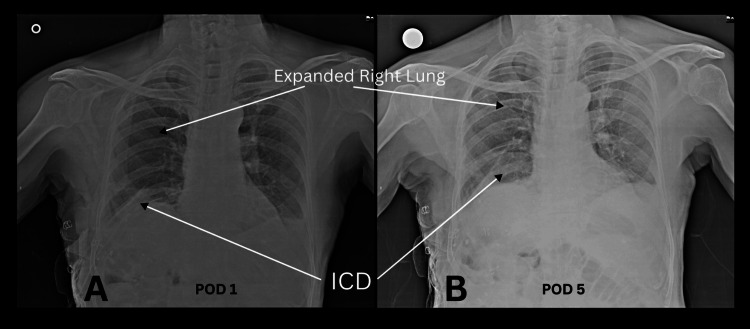
Chest X-ray on A) postoperative day 1, B) postoperative day 5.

A 78-year-old man with no significant comorbidities presented with symptoms of dyspnea on exertion, increased breathlessness, and right-sided chest pain persisting for three weeks. He had a history of excessive coughing due to a recent COVID-19 infection. Chest X-ray revealed air-fluid levels and a soft-tissue shadow in the right hemithorax. Computed tomography (CT) of the chest and abdomen showed right DE, with migration of the right lobe of the liver, gallbladder, and hepatic flexure of the colon into the right hemithorax, accompanied by right lung collapse. After completing all preoperative investigations, the patient was taken for surgery. A double-lumen endotracheal tube was inserted under general anesthesia, and laparoscopic ports were placed on establishing pneumoperitoneum. Intraoperative findings revealed that the right side of the diaphragm was stretched and thinned (Figure 1A), with two defects: one measuring 3 cm × 5 cm and another posteriorly measuring approximately 5 cm × 7 cm (two defects in Figure 1B). These defects caused herniation of the right liver lobe, gallbladder, hepatic flexure of the colon, duodenum, distal stomach, and omentum into the right hemithorax. Adhesiolysis was performed to reduce the abdominal contents back into the abdominal cavity. The right triangular ligament of the liver was divided to facilitate liver mobilization and prevent retraction into the thoracic cavity. The diaphragm defects were repaired using non-absorbable Ethibond (polyester) 2-0 sutures (Figure 1C), and a 15 cm × 20 cm composite mesh was placed and secured with tackers laterally and sutures medially (Figure 1D). After closing the abdomen, the patient was repositioned to the left lateral position, and thoracoscopic ports were inserted with single-lung ventilation. Due to the thinned diaphragm, thoracoscopic visualization was performed. Further diaphragm plication was conducted using non-absorbable Ethibond (polyester) 2-0 sutures (Figure 1E). Considering the large defect size, the risk of recurrence, and the patient’s age, an additional 15 cm × 15 cm lightweight polypropylene mesh was placed and secured with sutures (Figure 1F). A right intercostal drain was inserted. The total operative time was approximately 160 minutes, with an estimated blood loss of approximately 150 ml. Postoperative period was uneventful and with satisfactory lung expansion. Chest X-rays on postoperative days 1 (Figure 2A) and 5 (Figure 2B) demonstrated complete lung expansion. The patient was discharged on postoperative day 5 and was asymptomatic during follow-up visits up to one year.

Case 2

**Figure 3:**
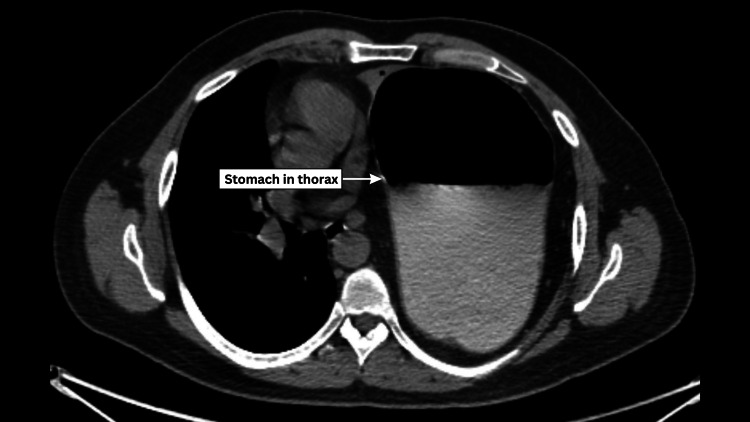
Preoperative axial view of the CT scan showing the whole of the stomach in the thorax

**Figure 4:**
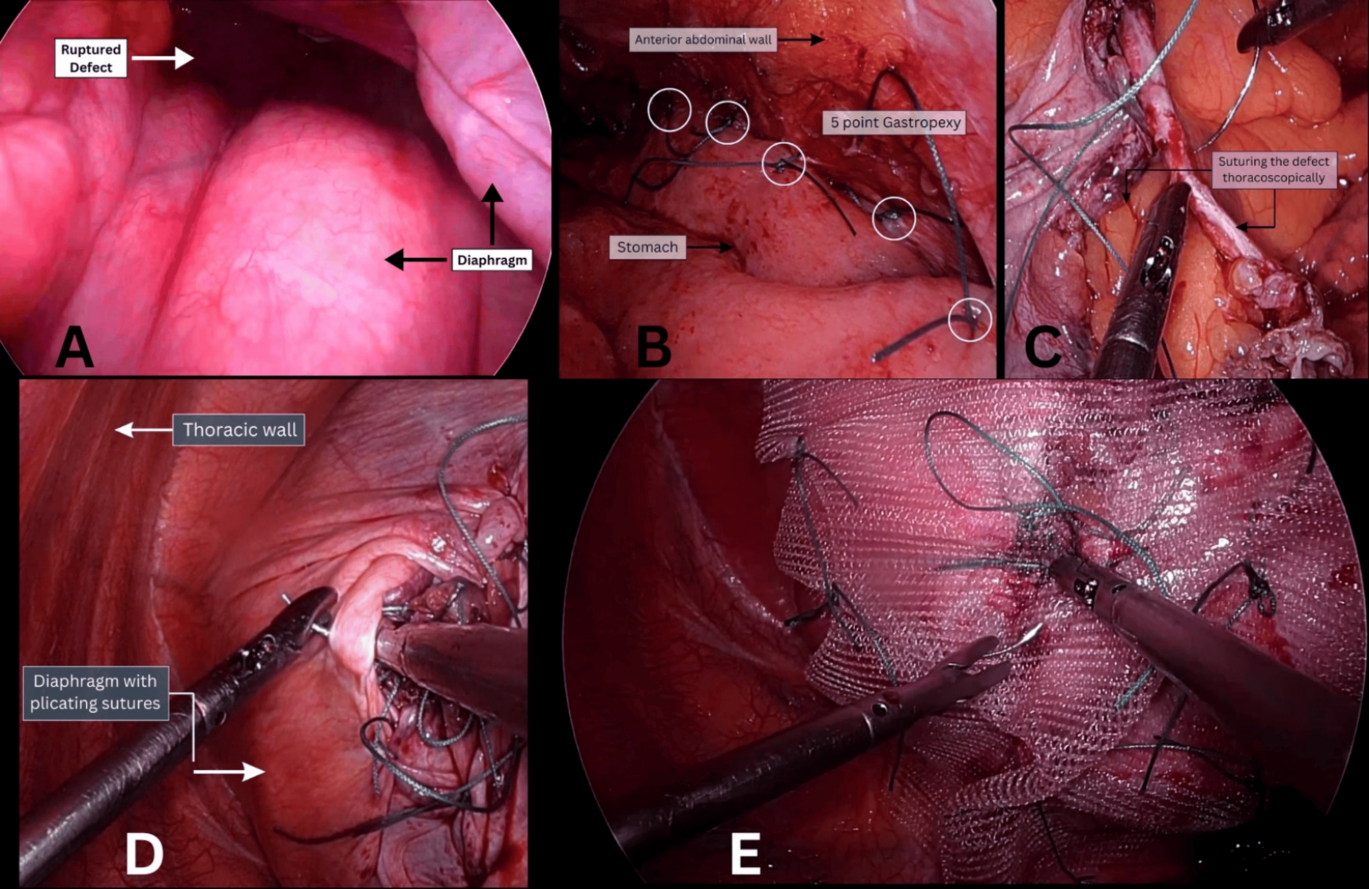
A) Laproscopic view of diaphragm defect; B) five-point gastropexy; C) thoracoscopic repair of defect; D) thoracoscopic plication of the diaphragm; E) reinforcement with the mesh.

**Figure 5:**
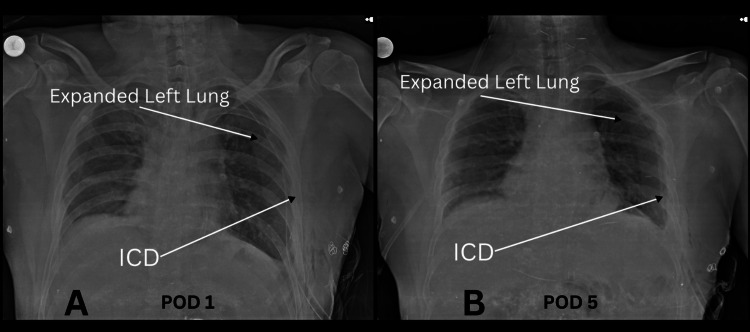
Chest X-ray on A) postoperative day 1; B) postoperative day 5.

A 37-year-old man with no significant comorbidities presented with breathlessness, upper abdominal pain, vomiting, and chest pain for two days. He had previously undergone Laparoscopic diaphragmatic plication for eventration in January 2023. Diagnostic imaging revealed air-fluid levels and a soft-tissue shadow in the left hemithorax on chest X-ray. A subsequent CT scan of the chest and abdomen identified a 6 cm × 6 cm defect in the postero-inferior aspect of the left diaphragm, accompanied by herniation of the fundus, body, and antrum of the stomach, along with omentum, and a potential organo-axial volvulus of the stomach [Figure 3]. After completing all preoperative investigations, the patient was prepared for surgical intervention. A double-lumen endotracheal tube was inserted under general anesthesia, and laparoscopic ports were placed to create pneumoperitoneum. During the operation, extensive omental adhesions to the diaphragm were observed, which required adhesiolysis. The omentum and stomach were reduced and repositioned into the abdominal cavity. A 6 cm × 5 cm defect in the postero-inferior aspect of the left hemidiaphragm was identified (Figure 4A), along with adhesion to the left lung. The vascularity of the stomach was assessed using intravenous indocyanine green, which confirmed normal perfusion. A five-point gastropexy was performed (Figure 4B). Given the condition of the diaphragm, which was noted to be exceptionally thin, a decision was made to proceed with thoracoscopic repair and plication. The defect was repaired thoracoscopically using Ethibond (polyester) 2-0 sutures (Figure 4C), followed by diaphragm plication (Figure 4D). To address the thinness of the diaphragm and reduce the risk of recurrence, a 15 cm × 15 cm soft polypropylene mesh was placed for reinforcement and secured with sutures (Figure 4E). Hemostasis was achieved, and an intercostal drain was placed. On postoperative day 1 (Figure 5A), the left lung had expanded effectively, and the postoperative period proceeded without complications. The patient was discharged on postoperative day 5 (Figure 5B) and remained asymptomatic during subsequent follow-up visits for up to one year.

## Discussion

During contraction, the diaphragm pulls down on its central tendon, increasing the vertical diameter of the thoracic cavity. This downward movement causes the diaphragm to flatten and push into the abdominal cavity, creating negative pressure that draws air into the lungs. Concurrently, the external intercostal muscles, situated between the ribs, elevate the anterior chest wall, expanding the chest cavity in a manner analogous to raising the handles of a bucket. During exhalation, the chest wall and rib cage return to their original positions, and the diaphragm relaxes and rises, expelling air from the lungs. DE alters normal respiratory physiology, leading to complications such as atelectasis, ventilation-perfusion mismatch, mediastinal shift to the opposite side, diminished pulmonary spirometry, paradoxical movement of the affected diaphragm segment, and altered pulmonary blood flow distribution [3-4].

The primary symptom experienced by most patients is dyspnea. Diaphragmatic plication aims to reduce the surface area of the redundant diaphragm by folding it to a more physiologic and normal position, thereby improving pulmonary function and diaphragm movement during respiration. Protection of the phrenic nerve is crucial during this procedure. Unilateral plication typically results in a 30% increase in trans-diaphragmatic pressure, enhancing the diaphragm's overall muscular force [5-6]. Post-plication, tidal volume improvements, ventilator rate reduction, and forced expiratory volume can alleviate symptoms [7].

Surgery is not indicated solely based on elevated hemidiaphragm or paradoxical movement without significant dyspnea. Three plication techniques, i.e., "reefing the mainsail," "invaginating the diaphragmatic dome," and "pleating," have demonstrated satisfactory outcomes [8].

DE generally has a better prognosis compared to diaphragmatic hernias, with lower rates of postoperative morbidity and mortality. Diaphragmatic plication is a relatively straightforward procedure with fewer complications during the initial postoperative period and favorable long-term results. Complications such as pneumothorax, recurrence, or rupture of the attenuated diaphragm are rare. However, phrenic nerve and vascular damage during plication can lead to diaphragm ischemia, atrophy, and necrosis, increasing the risk of rupture. A weakened diaphragm may rupture under elevated intra-abdominal pressure, potentially leading to volvulus due to negative intrathoracic pressure (as in case 2) [9].

Calcified or nonpliable diaphragms, certain neuromuscular disorders, and morbid obesity are relative contraindications for plication. Morbidly obese patients should ideally undergo bariatric evaluation before plication, as significant weight loss may alleviate dyspnea and negate the need for surgical intervention [10]. Suturing large defects strengthens the diaphragm and provides a suitable surface for mesh placement, preventing extrusion. Reinforcing sutured defects with mesh is recommended to improve the physiological response and support the defect [11].

Laparoscopic plication reduces intercostal nerve pain, avoids single-lung ventilation, improves working space, and enhances abdominal organ visualization, thus reducing injury risk. For left-sided DE, laparoscopic techniques may reveal additional potential combined procedures, such as gastric fundoplication. However, laparoscopic access may sometimes require a diaphragm incision, which has drawbacks [12].

The thoracoscopic approach is more commonly reported in the literature than the laparoscopic method. We have our previous publications, AC Oak et al. describing various minimal access techniques used in the plication of the diaphragm [13] and Gupta et al. reporting the first case of right-sided ruptured eventration [14]. The benefits of thoracoscopic techniques include better control of thoracic adhesions, less interference from abdominal organs, and direct visualization of the phrenic nerve, which aids in its preservation. Although some studies note chronic pain post-thoracoscopic surgery, this is manageable with modern analgesics and blocks. The thoracoscopic method is particularly recommended for right-sided DE due to the minimal risk of bowel injury as the liver is situated beneath the right subphrenic, and there is very little chance that the bowel will be harmed during the primary closure and plication of the diaphragm. However, the subphrenic liver would become a major obstacle in the laparoscopic approach. Gupta A et al. [15] mentioned an increase in recurrence with the thoracoscopic approach, but most other studies mention no difference in laparoscopic and thoracoscopic approaches [16].

Endostaples are increasingly used for diaphragm resection and plication. However, they have been associated with a higher recurrence rate, as mentioned in Miyeong et al.'s study [17], whereas Balamurugan et al.'s study [18] mentions that plication performed with staplers and sutures demonstrates both safety and efficacy in treating DE. Recent advancements in staple technology, such as the introduction of three-layer staples, have shown promise, although the efficacy of traditional plication methods remains debated.

In our report, we have performed thoracoscopic plication with mesh reinforcement in both cases. This technique has been previously documented as safe by Kim et al. [19].

It is important to position sutures parallel to the phrenic nerve branches during plication to avoid damage. Avoid full-thickness sutures to prevent damage to structures on the opposite side of the diaphragm. If the diaphragm is thin and attenuated, mesh reinforcement may be needed. Over-plication can result in excessive tension, which may lead to lateral avulsion of the diaphragm.

## Conclusions

This report presents two rare cases: the first involves a right-sided ruptured DE, while the second concerns a ruptured diaphragm following laparoscopic plication of the DE. Plication is the standard treatment for DE. Both laparoscopic and thoracoscopic approaches are feasible and safe, offering distinct advantages and disadvantages. In cases involving a papery-thin diaphragm, mesh reinforcement is necessary to prevent recurrence; existing literature also supports this practice. The use of tackers for plication remains a subject of debate due to concerns regarding potential recurrence. It is essential to exercise caution to avoid over-tightening during the plication process, as this may lead to diaphragm rupture. Effective management of DE necessitates meticulous surgical technique and careful consideration of patient-specific factors to optimize surgical outcomes. Surgical repair is indicated in symptomatic cases, and the choice of technique should be tailored to the surgeon’s expertise and the individual needs of the patient.
